# Supplementary material for: Histological and Immunohistochemical Characterization of Osteoimmunological Processes in Scaffold-Guided Bone Regeneration in an Ovine Large Segmental Defect Model
Source: Biomedicines. 2023 Oct 13;11(10):2781. doi: 10.3390/biomedicines11102781 (PMC10604530; doi:10.3390/biomedicines11102781)
Supplement: Supplementary file 1 [file biomedicines-11-02781-s001.zip › biomedicines-2596602-supplementary.pdf]

Histological and Immunohistochemical Characterization of Osteoimmunological Processes in Scaffold-Guided Bone Regeneration in an Ovine Large Segmental Defect Model

Ronja Finze [1,2,‡], Markus Laubach [1,3,4,‡], Mairim Russo Serafini [5], Ulrich Kneser [2], Flavia Medeiros Savi [1,3,6,*]

1 Supplementary Table 1. Details of experimental groups.

Details of experimental groups. mPCL-TCP = medical-grade polycaprolactone and tricalcium phosphate; mPCL-TCP-CaP = medical-grade polycaprolactone and tricalcium phosphate and calcium phosphate surface coating; PRP = Platelet-Rich-Plasma; ICGB = iliac crest bone graft; RIA system = Reamer-Irrigator-Aspirator system; rhBMP-7 = recombinant human bone morphogenic protein-7; CPF = cortico-periosteal flap; Allo-MSC = allogenic mesenchymal stem cells

Experimental Groups			
Scaffold	Bone Graft Material / Substitute	Tibial Defect Sizes	Time Points
mPCL-TCP	RIA	6 cm	6 hours<br>12 months<br>14 months<br>15 months<br>21 months<br>27 months
mPCL-TCP	ICGB (6 ml)	6 cm	2 months<br>6 months
mPCL-TCP	rhBMP-7 (2 mg) carried in PRP	6 cm	12 months
mPCL-TCP-CaP	CPF	3 cm<br>6 cm	12 months<br>12 months<br>15 months
mPCL-TCP	Allo-MSC	3 cm	36 months

2 Supplementary Table 2. Antibody markers used for immunohistochemistry.

Verified primary antibody staining to detect inflammatory and blood vessel markers as well as bone ECM proteins in the preclinical critical-sized bone defect model. PK: Proteinase K. ab: abcam; SC: Santa Cruz;

Antibody	Positive control	Reactivity	Raised in	Clonality	Cat. no.	Antigen retrieval	Blocking	Dilution	Incubation	DAB
**Inflammation/Osteoclastic activity markers**										
Nitric oxide synthase (iNOS)	Sheep spleen	Mouse	Rabbit	Polyclonal	ab15323	PK 5 min	2% BSA	1:100	60 min	3 min
Mannose receptor (MR)	Sheep liver	Human, Mouse, Rat	Rabbit	Polyclonal	ab64693	PK 5 min	2% BSA	1:500	60 min	45 sec
Arginase-1 (ARG-1)	Sheep spleen	Human, Mouse, Rat	Rabbit	Polyclonal	Proteintech 16001-1-AP	PK 5 min	2% BSA	1:100	60 min	5 min
Cluster of differentiation 68 (CD68)	Sheep spleen	Mouse, Rat	Rabbit	Polyclonal	ab125212	PK 5 min	2% BSA	1:300	60 min	1:30 min
Interferon regulatory factor 5 (IRF5)	Sheep spleen	Human, Mouse, Rat,<br>Human, Mouse, Rat	Mouse<br>Rabbit	Monoclonal<br>Polyclonal	sc-56714<br>Proteintech 10547-1AP	PK 5 min<br>PK 5 min	2% BSA<br>2% BSA	1:100<br>1:100	60 min<br>60 min	5 min<br>3 min
Cluster of differentiation (CD3)	Sheep spleen	Human	Mouse	Monoclonal	ab17143	PK 5 min	2% BSA	1:100	60 min	5 min
Cluster of differentiation 45 (CD45)	Sheep spleen	Human, Mouse, Rat	Rabbit	Polyclonal	ab10558	PK 5 min	2% BSA	1:100	60 min	5 min
**Vascularization markers**										
Vascular endothelial growth factor (VEGF)	Sheep tibia	Human, Mouse, Rat	Rabbit	Polyclonal	SC152	PK 5 min	2% BSA	1:500	60 min	1:30 min

Cluster of differentiation 31 (CD31) (PECAM-1 (M-20))	Sheep tibia	Human, Mouse, Rat	Goat	Polyclonal	SC1506-R	PK 5min	2% BSA	1:1000	60 min	1 min
von Willebrand factor (vWF)	Sheep tibia	Human	Rabbit	Polyclonal	IR527	PK 5min	2% BSA	Ready to use	60 min	1:30 min
Alpha smooth muscle actin (α-SMA)	Heart	Human, Mouse, Pig, Rabbit, Rat, Sheep,	Mouse	Monoclonal	ab7817	PK 5 min	2% BSA	1:500	60 min	2 min
Angiopoietin-1 (ANG1)	Sheep tibia	Human, Mouse, Rat	Rabbit	Polyclonal	ab102015	PK 5 min	2% BSA	1:100	60 min	1 min
Noggin (NOG)	Sheep tibia	Human, Mouse, Rat	Rabbit	Polyclonal	SC25656	PK 5 min	2% BSA	1:100	60 min	5 min
NOTCH 1	Sheep tibia	Human, Mouse, Rat	Rabbit	Polyclonal	SC6014-R	PK 5 min	2% BSA	1:100	60 min	5 min
**Extracellular matrix markers**										
Collagen type I (COL I)	Sheep tibia	Human<br>Human	Rabbit<br>Rabbit	Polyclonal<br>Monoclonal	ab34710<br>ab138492	PK 5min	2% BSA	1:1000<br>1:500	60 min	2 min<br>30 sec
Collagen type II (COL II)	Sheep tibia	Avian, Bovine, broad species, Chicken, Fish, Goat, Human, Mouse, Ovine, Rabbit, Rat, Shark, Zebrafish, Xenopus	Mouse	Monoclonal	DSHB<br>II-II6B3	PK 5min	2% BSA	1:100	60 min	40 sec
Bone morphogenetic protein 2 (BMP2)	Sheep tibia	Human, Mouse, Rat,	Mouse	Monoclonal	SC137087	PK 5 min	2% BSA	1:50	60 min	overnight

Osteoprotegerin (OPG)	Sheep tibia	Human	Rabbit	Polyclonal	ab73400	PK 5 min	2% BSA	1:100	60 min	30 sec
Alkaline Phosphatase (ALP)	Sheep tibia	Human, Mouse, Rat,	Mouse	Monoclonal	sc166261	PK 5 min	2% BSA	1:500	60 min	5 min
Sclerostin (SCL)	Sheep tibia	Human, Mouse, Rat, Sheep,	Rabbit	Polyclonal	ab63097	PK 5 min	2% BSA	1:100	60 min	1 min
Osteomodulin (OMD)	Sheep tibia	Human, Mouse	Rabbit	Polyclonal	ab154249	PK 5 min	2% BSA	1:500	60 min	1 min
Osteonectin (ON)	Sheep tibia	Bovine, Cow, Dog, Human, Monkey, Pig, Rabbit, Rat	Mouse	Monoclonal	DSHB - AON-1	PK 5 min	2% BSA	1:100	60 min	2 min
Osteopontin (OPN)	Sheep tibia	Dog, Human, Mouse, Pig, Rat,	Rabbit	Polyclonal	ab8448	PK 5 min	2% BSA	1:100	60 min	40 sec
Osteocalcin (OC)	Sheep tibia	Cow, Dog, Goat, Human, Pig, Rat, Rabbit, Sheep	Mouse	Monoclonal	ab13418	PK 5 min	2% BSA	1:500	60 min	30 sec

1. **Supplementary figure 1. Schematic overview of the histologic sectioning methodology used in our model of the ovine segmental tibial defect.**

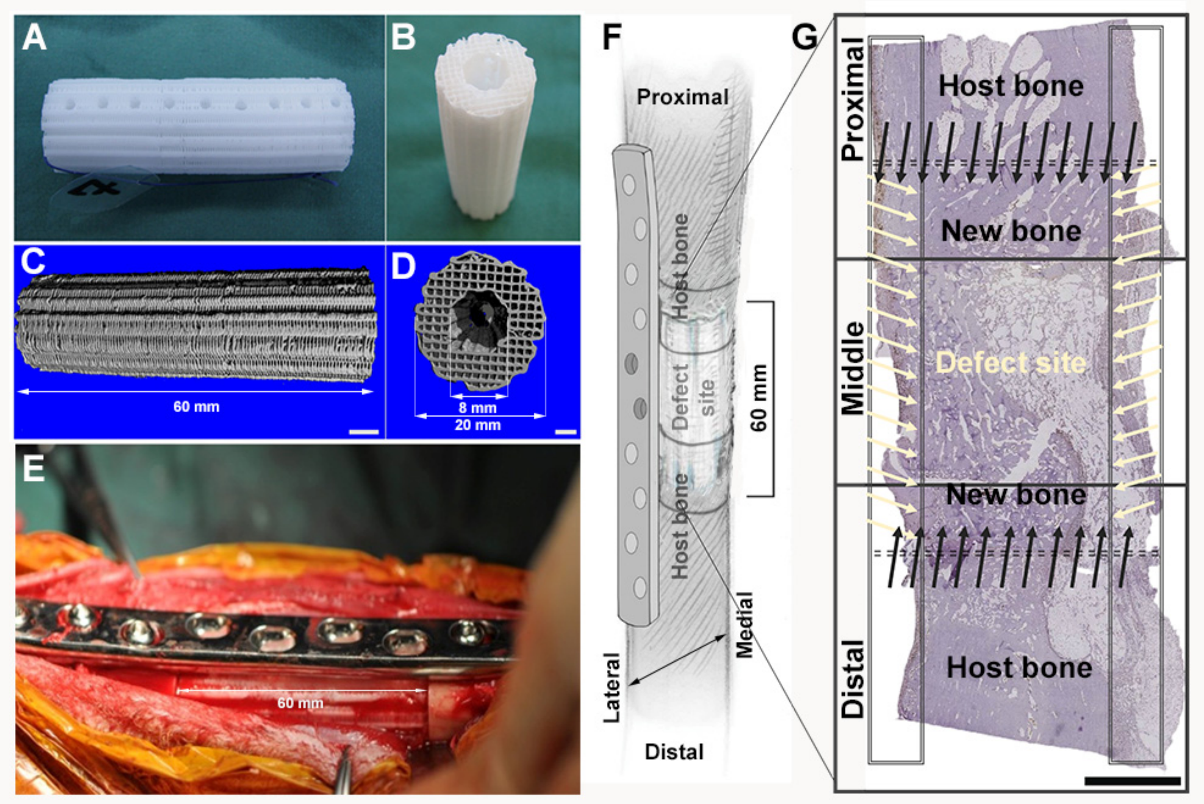

Supplementary figure 1. (A-D) Longitudinal and radial view, and dimensions of the mPCL-TCP scaffold**.** (F-G) Standardized transverse sectioning planes for the immunohistological specimens are proximal host bone and defect site interface, middle: defect site only; distal host bone and defect site interface. Reproduced and modified from Henkel *et al*. [20] and Medeiros Savi *et al*. [78]. Scale bar: 5 mm.

2. Supplementary figure 2. Immunohistochemical analysis of protein expression in SGBR throughout the entire bone regeneration period, negative controls.

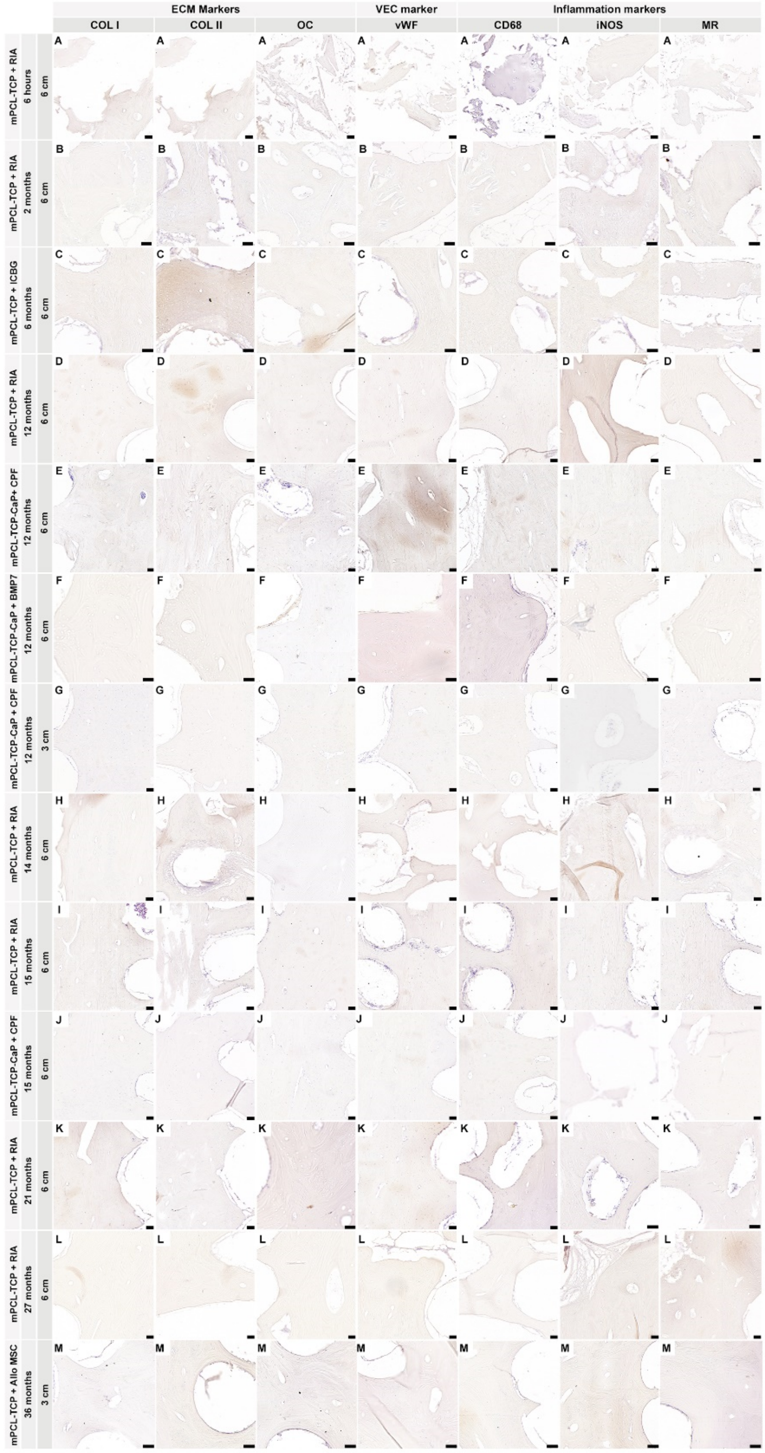

Supplementary figure 3. Immunohistochemical analysis of protein expression in SGBR throughout the entire bone regeneration period. Negative controls for Collagen type I (Col I), Collagen type II (Coll II); Osteocalcin (OC); von Willebrand Factor (vWF); cluster of differentiation 68 (CD68); inducible nitric oxidase synthesis (iNOS); Mannose receptor (MR). Scale bars: 50µm

3. **Supplementary figure 3. Inflammation markers negative controls.**

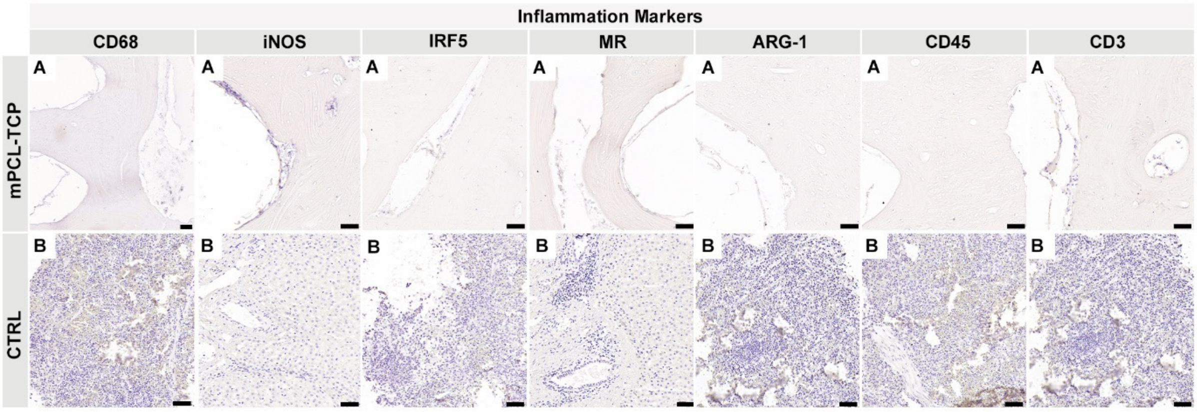

Supplementary figure 4. Inflammation markers negative controls for Cluster of differentiation 68 (CD68), Nitric oxide synthase (iNOS), interferon regulatory factor 5 (IRF5), mannose receptor (MR), Arginase-1 (ARG-1), cluster of differentiation 45 (CD45) and cluster of differentiation 3 (CD3). Scale bars: 50µm

4. **Supplementary Figure 4. Vascular endothelial cellular markers negative controls.**

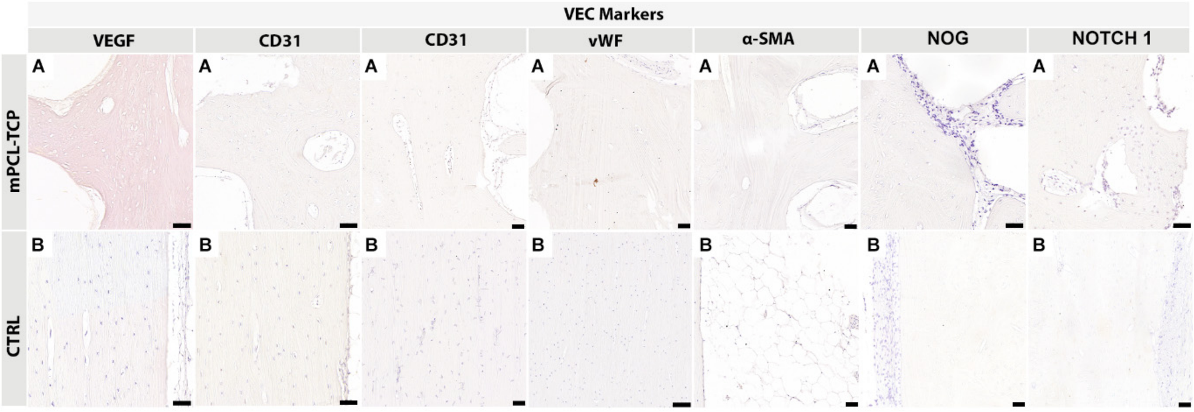

Supplementary figure 5. Vascular endothelial cellular markers negative controls for Vascular Endothelial Factor (VEGF); Angiopoietin (ANG1); Cluster of differentiation 31 (CD31); von

Willebrand Factor (vWF); Anti smooth Muscle Actin (α-SMA); Noggin (NOG). Scale bars: 50µm

5. Supplementary figure 5. Extracellular matrix markers negative controls.

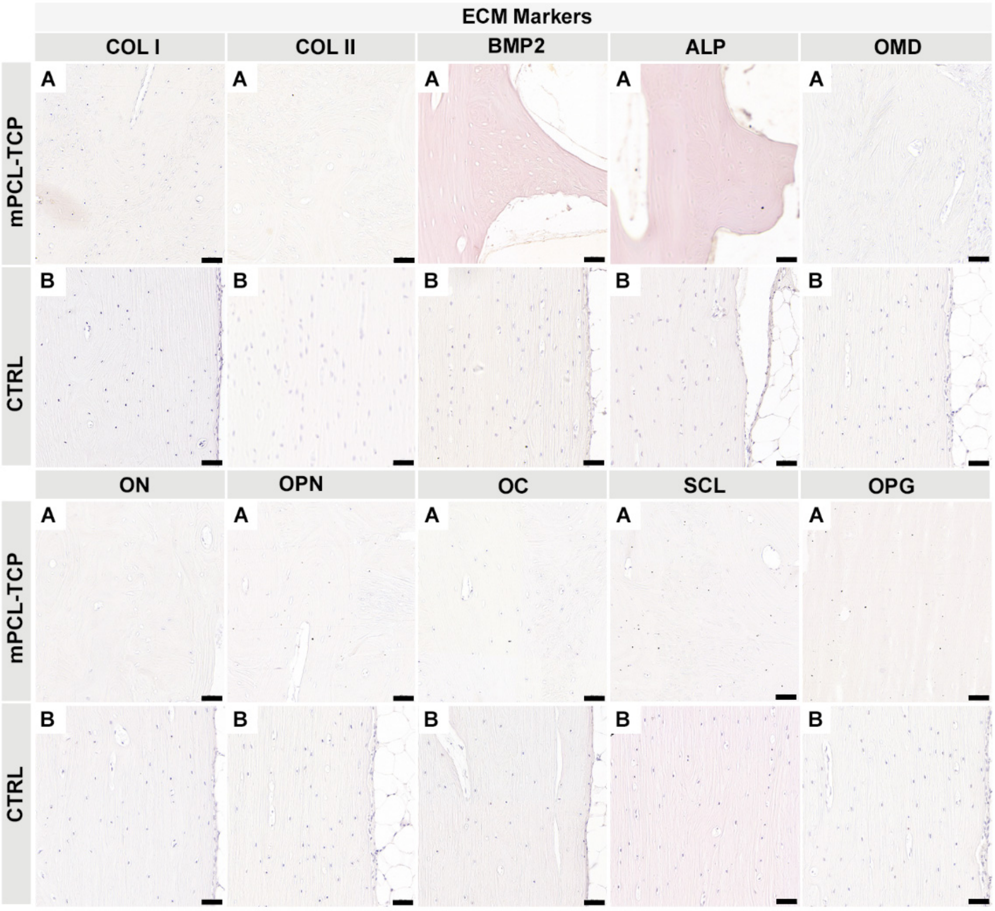

Supplementary figure 6. Extracellular matrix markers negative controls for: Collagen type I (Col I), Collagen type II (Coll II); Bone Morphogenic Growth Factor 2 (BMP2); Alkaline Phosphatase (ALP); Osteomodulin (OMD); Osteonectin (ON); Osteopontin (OPN); Osteocalcin (OC); Sclerostin (SCL) and Osteoprotegerin (OPG); Isotypes controls for NOG and NOTCH were used from another tissue sample, however done at the same time. Scale bars: 50µm
